# Family functioning and satisfaction: A comparative study between hookah users and non-users

**DOI:** 10.4102/phcfm.v11i1.2095

**Published:** 2019-11-12

**Authors:** Nicolette Roman, Edna Rich, Charl Davids, Fatiema Benjamin, Matthew Taylor

**Affiliations:** 1 Child and Family Studies, Department of Social Work, University of the Western Cape, Bellville, South Africa; 2 Centre for Student Counselling and Development, Stellenbosch University, Stellenbosch, South Africa; 3 Department of Psychological Sciences, University of Missouri, St. Louis, United States

**Keywords:** family functioning, family satisfaction, hookah users, hookah non-users, family context

## Abstract

**Background:**

Although there has been an exponential growth in hookah use on a global scale, research within the context of South Africa is very limited. While hookah use is known internationally to be a health and addiction risk, the focus is on university students and not on families.

**Aim:**

This study aims to compare the family functioning and family satisfaction among hookah users and non-users.

**Setting:**

This study was conducted in low or middle-high class socio-economic status areas of Cape Town, South Africa.

**Methods:**

A quantitative method was employed to test for significant differences with a sample of 1193 participants, in which each participant represented a family. An independent t-test was used to test for significant differences between hookah users and non-users.

**Results:**

One-third (34%) of the participants indicated that they smoked hookah pipe, with the general age of onset being 16.5 years. In trying to understand the family context, it was found that 28% of hookah users indicated that the hookah pipe was used as a means of socialising with others in the family, and 24% of parents indicated that they were more accepting of family members smoking the hookah pipe. Findings also suggest that families of hookah users have less cohesion, expressiveness and family satisfaction, and more conflict and permissiveness than families of non-users.

**Conclusion:**

This study provides and extends knowledge regarding the family in hookah pipe use. This information could assist in reducing hookah pipe use, and building healthier and more resilient communities by formulating prevention and intervention strategies to reduce hookah use

## Introduction

Hookah pipe is understood to be a cultural or traditional phenomenon^1^ as it has originally stemmed from Middle Eastern countries and is rooted in certain cultures.^2^ However, hookah being used in hookah bars, cafés and restaurants,^3^ it has become more of a social phenomenon. It could be that with the raised awareness of the risk of cigarette smoking, the use of hookah pipe has become an alternative to cigarette smoking,^4,5,6^ as it is socially understood to be less harmful.

Globally, studies on hookah pipe consider it to be a health hazard and public health concern^7,8^ and view it as a gateway for other substances.^8,9^ There has been an exponential growth in its use on a global scale^10^ with hookah popularity and social acceptance being attributed to the factors such as easy availability, attractive designs and various flavoured fragrant tobacco variants used in hookahs, called *maassel*.^10,11^

According to a review conducted by the World Health Organisation (WHO),^1^ studies focusing on hookah pipe have been mainly conducted among university students, the health risks of using hookah pipe and the prevalence of using hookah pipe across the world. The prevalence of using hookah among university students has steadily increased globally over the past 10 years, especially amongst western countries.^12,13,14^ The health risks identified included, amongst others, different forms of cancer, tuberculosis, lung diseases and heart disease.

Family factors such as monitoring, parent – adolescent closeness and consistent discipline have been identified as protective factors against adolescents smoking cigarettes.^15^ However, negative family interactions, for example poor parental monitoring,^16^ cohesion and family conflict, have been identified as contributing factors to adolescent tobacco use.^17,18^ As such, previous research indicated that family values and behaviour in relation to acceptability of hookah use could be a risk factor for the onset and increased use of hookah pipe.^9,19,20^ This is related to its social acceptability. These studies do not however present information regarding the family factors, such as family practices of hookah pipe users. For example, research shows that family conflict, cohesion, expressiveness and permissiveness play a role in child/adolescent tobacco and alcohol use.^17,18^ Therefore, family factors are important when looking at adolescent substance practices.

These research outcomes were, however, not previously presented in the field of hookah pipe use. Thus, this study proposes that there are significant differences between families of hookah pipe users and non-users with particular focus on familial conflict, cohesion, expressiveness and permissiveness.

## Methods

### Study design

This study used a quantitative observational cross-sectional analytical design. This is related to the study being aimed at comparing hookah users and non-users in terms of family functioning and family satisfaction.

### Setting

The intention for this study was to obtain a heterogeneous sample from low and middle-high socio-economic areas. The economic criterion used for stratification in this study was based on the location of the area. That said, data were collected across townships, the Cape Flats and the northern suburbs of Cape Town, South Africa. These areas were randomly selected based on the previous geographical and socio-economic dispensations which took place during apartheid in the country. These areas clearly highlight the status of race as well as socio-economic status (SES). The township area used in this study is bordered by Jan Smuts Drive to the west and two separate highways, one to the south and another to the east. Additionally, data were collected from the area located east of the city centre on the Cape Flats and south of the highway. The area predominantly serves as a residential area but also comprises a railway station, stadium as well as industrial and commercial zones. Furthermore, the suburb in which data were collected forms a part of the greater Cape Town metropolitan area and is surrounded by farms producing wine and wheat.

### Study population and sampling strategy

Study participants were recruited by means of systematic and convenience sampling. Initially, a systematic ‘door-to-door’ sampling or recruitment process was used, which was adapted from the process shown by Flynn et al.^21^ Every fifth house from the starting point was approached in the recruitment process. However, once data collection started, the systematic sampling approach deemed challenging due to people not being at home or unwilling to participate. The researchers then began the process of convenience sampling by randomly selecting participants in the same street and/or community. This is related to researchers accessing participants based on their availability. The reason for these sampling techniques as deemed appropriate was the time period set aside for data collection, as data were to be collected within 2–3 months. Additionally, individuals who agreed to be a part of the study represented an entire family. Therefore, one participant represented an entire household. Participants were included in the study if they were the parent of at least one child aged between 0 and 17 years. This includes both parents. Furthermore, individuals were excluded from the study if they were secondary caregivers to a child, such as a grandparents or other family member, or had children aged 18 years and older. In low socio-economic environments, fieldworkers involved with these communities were used. These fieldworkers were informed to approach and invite a participant from every fifth family from their starting point in the identified areas. Students were used as fieldworkers in middle-high socio-economic areas, with the same sampling strategy applied by selecting every fifth family from their starting point. This sampling strategy was used to ensure that fieldworkers were not biased in their selection of participants as well as frequent debriefing between fieldworkers and the primary investigator to minimise bias.

### Data collection

The questionnaire used was compiled to address family functioning and family satisfaction among hookah users and non-users. Demographical information such as gender, age, education level and family structure as well as a section that focused on hookah use in the family was included in the questionnaire. Furthermore, two scales, Satisfaction with Family Life Scale and Family Functioning (the Family Assessment Device) were used to understand family functioning and family satisfaction of the participants.

#### Satisfaction with Family Life Scale

This Likert scale was adapted from the Satisfaction with Life Scale, which measures global life satisfaction as understood in terms of an individual’s overall assessment of his or her quality of life.^9^ In this particular study, it is a perception of subjective wellbeing based on an individual’s perception based on his or her family life. This scale has shown sound structural and convergent validity within the context of South Africa with a reliability coefficient (α = 0.79).^22^

#### Family functioning (the Family Assessment Device)

Roman et al.9 identified this Likert scale as being developed to assess a family based on various factors. These factors include communication, problem-solving, behaviour control, affective involvement, affective responses and roles. The reliability coefficient for this scale has shown to be acceptable with a Cronbach’s α of 0.78.^22^

All fieldworkers were trained in the data collection process and the use of the data collection instrument before the implementation of this process. Fieldworkers approached and invited an adult family member to voluntarily participate in the study. Before these individuals accepted participation in the study, they were provided with an information sheet and consent form, which was explained to them by the fieldworker.

### Data analysis

The raw data were double-entered into the Statistical Program for Social Science (SPSS) and coded and cleaned in order to check for errors. Data analysis included descriptive statistics and inferential statistics. The inferential statistics used were independent t-tests to test significant differences concerning family functioning and family satisfaction among hookah users and non-users.

### Ethical considerations

On receiving ethics clearance and the commencement of data collection, researchers sought informed consent from all participants. This consisted of participants being presented with information pertaining to the details of the study and assuring that all information obtained from them would be confidential and they have the right to withdraw from the study at any time without any consequence. Participation was completely voluntary. Furthermore, no harm came to the participants during the data collection process.

Ethical approval for the study was obtained from the Humanities and Social Science Research Ethics Committee of the University of the Western Cape (Reference number: HS/16/3/48).

## Results

### Descriptive results

The profile of the final sample predominantly comprised the following: female participants: 54%, English-speaking participants: 50%, average age of participants: 33 years, education level of participants: Grade 11 and employed participants: 54.7%. Based on the original issue of 1400 questionnaires, the return rate for the current study was 85.21%. Therefore, the final sample comprised 1193 participants, each participant representing a family of which 72% were from low SES areas and 28% from middle-high SES areas. The difference in sample size for the low and middle-high SES areas was mainly caused by lack of participation in the middle-high SES area.

Overall, the current study found that 34% of participants indicated smoking of hookah pipe in which the age of onset was 16.5 years ($\bar{X}$ = 16.48). Majority of the participants (37%) who responded to the question related to hookah pipe use were from low SES areas as opposed to middle-high SES areas (26%).

### The family context and hookah use in the family

In the current study, it was found that various substances were used in the family. These substances include marijuana, also known as dagga (21%); mandrax (3%); heroine (2%); cocaine (4%); alcohol (49%); tik (7%) and glue (2%). The following aspects were also found in the family: (1) knowledge of the harmful effects of hookah pipe (65%); (2) hookah is predominantly combined with alcohol (30%); (3) hookah pipe is mostly used at parties (72%); (4) hookah use in the family (60%) and (5) acceptability of use in the family (24%). A substantial proportion of participants (17%) indicated that children were present when hookah pipe was used in the family. This study also found that children as young as 2–6 years (3%) use hookah pipe. As previously indicated, one participant represented the entire household. That said, when focusing on hookah use in the family, 62% of the participants stated that hookah pipe was not used as a means of socialising in the home whereas 28% indicated that hookah pipe was in fact used as a means of socialising n the family (see Table 1).

**TABLE 1:** Reasons for hookah use in the family.

Variable (*n*)	Yes		No		Don’t know *n*	%
	*n*	%	*n*	%		
Is hookah pipe used as a means of socializing in your family? (*N* = 891)	246	28	549	62	96	11
Is hookah pipe used as a means of communication between family members? (*N* = 886)	106	12	682	77	98	11
Do family members talk easily with each other when they are smoking hookah pipe? (*N* = 885)	141	16	571	65	173	20

Furthermore, participants were asked about parents’ acceptance pertaining to smoking the hookah pipe. Although the majority indicated that the hookah pipe use was not accepted in the family, those who indicated its acceptance stated that parents were more consenting of their own family members using the hookah pipe in the family (24%) than non-family members (20%). This means that parents were accepting of their own family members smoking hookah pipe in the home as compared to non-family members such as friends.

### Family functioning and family satisfaction

The manner how the family functions in terms of cohesion, conflict, expressiveness, permissiveness and being satisfied is as follows. The results shown in Table 2 suggest that when families are cohesive (*r* = 0.53; *r*^2^ = 0.28) and expressive (*r* = 0.47; *r*^2^ = 0.16), family members are be more satisfied with others in the family. This suggests that cohesion in the family accounts for 28% and expressiveness for 22% towards family satisfaction. In addition, results also suggest that if conflict (*r* = −0.40; *r*^2^ = 0.16) and permissiveness (*r* = −0.19; *r*^2^ = 0.04) are present in the family, its members are less satisfied with others in the family. This is associated with conflict in the family accounting for 16% and permissiveness accounting for 4% towards family satisfaction. These results are related to the study being focused on family function and family satisfaction between hookah users and non-users.

**TABLE 2:** Associations between different variables.

Variables	Family satisfaction	*r*^2^
1. Conflict in the family	−0.40**	0.04
2. Cohesion in the family	0.53**	0.28
3. Expressiveness in the family	0.47**	0.16
4. Permissiveness in the family	−0.19**	0.22

** , *p* < 0.05, 2-tailed.

**TABLE 3:** Hookah use group differences: Family functioning and family satisfaction.

Variable	Hookah users Mean	s.d.	Hookah non-users Mean	s.d.	*p*
Conflict	2.28	0.52	2.12	0.53	< 0.001
Cohesion	2.94	0.63	3.13	0.64	< 0.001
Expressiveness	2.74	0.66	2.93	0.62	< 0.001
Permissive	2.34	0.61	2.23	0.55	< 0.001
Family satisfaction	3.44	0.87	3.76	0.85	< 0.001

s.d., standard deviation.

The results in Table 2 show that there are significant differences between hookah users and non-users based on family functioning and being satisfied in the family. Hookah users were less satisfied in the family ($\bar{X}$ = 3.44), had more permissive family functioning (without rules and discipline) ($\bar{X}$ = 2.34) and had less expressiveness ($\bar{X}$ = 2.74) and cohesion ($\bar{X}$ = 2.94) in the family but greater conflict ($\bar{X}$ = 2.28). It was observed that family relationship was a challenge in the families of hookah users. These results provide insights into the potential risk factors of hookah use in a family.

## Discussion

The aim of this study was to compare family functioning and family satisfaction between hookah users and non-users. It was found that children aged between 2 and 6 years started using hookah pipe, with the general age of onset being 16.5 years. Additionally, differences were found in the way the family functions, in particular conflict in the family, cohesion, expressiveness and permissiveness, as well as family satisfaction in families of hookah users and non-users. This is related to the hookah users in the study reporting higher levels of conflict as well as less cohesion, expressiveness, permissiveness and family satisfaction in the family as opposed to the hookah non-users in the study.

### Prevalence

Establishing prevalence rates in the study was important. Prevalence rates indicate the extent of problem and are significant in implementing any sort of intervention or prevention strategy as it provides an indication of the individual having the problem, thus who should be the target group. Nationally, studies report rates of prevalence as between 18%^14^ and 60.9%.^23^

Of concern in the current study was the age of onset of smoking hookah, which was younger than indicated in the previous studies conducted in South Africa^9^ but slightly higher than as shown in international research,^23^ where majority of participants started smoking hookah pipe between 13 and 15 years of age. In line with the identified age of onset of hookah use was the prevalence of hookah use among children and adolescents. This finding suggests a similar prevalence of use as observed in a previous pilot study in terms of increasing age per category as well as use among young children. This then particularly becomes a public health concern, as young people who start smoking tobacco at an early age are at a risk of nicotine addiction as opposed to those who start later.^9^ Global research mainly reflects its use among adolescents^1^ and not younger children, which could be a phenomenon pertinent to South Africa, and specifically to the Western Cape.

### The role of the family

In this study, the family is situated as a context for use. Although parents are the ones who should be held accountable for the care and wellbeing of their children, families provide exposure and experience to child and adolescent to use hookah as indicated in the pilot study conducted by Roman et al.^9^ In particular, majority of the participants indicated that hookah pipe was not used as a means of socialising in the family; however, for a smaller percentage hookah was used in the family as a form of socialising, and was acceptable by family members. This is in line with the research which found that parents were more accepting of their children using hookah in the family, rather than going outside to use it.^24,25^ This is further supported by a qualitative study conducted in Iran which focused on the factors contributing to the first hookah use by women.^11^ In the stated study, an in-depth perspective was provided of the traditions of socialising in families in Iran by preparing and using hookah pipe. Previous research in other countries suggest that if one family member smokes hookah pipe, there is a stronger likelihood that children would follow.^20^ This is in line with the small percentage of children aged between 2 and 6 years who were found to smoke hookah pipe. This then becomes a blueprint for permissibility and acceptability from the family to the community to the society. Although for the majority of participants the hookah pipe was not used in the family home as a means of easier communication or socialising, it should be noted that in other families it enhanced family interactions.

### Family functioning and family satisfaction

This research is the first study to provide information regarding the status of families in terms of family functioning and family satisfaction among hookah users and non-users. Although family functioning is pertinent in the development of children and adolescents, and also in moderating what children are exposed to,^9^ this study found families’ ability to function effectively somewhat problematic. More specifically, it found that the families of hookah users had less cohesion, expressiveness and family satisfaction compared to families of non-users, in turn indicating that the families of hookah users experience more conflict and permissiveness in their homes. This could be related to these families’ likelihood of being exposed to violence, as well-functioning families were found to moderate exposure to and perpetration of community violence.^9^ Therefore, when comparing the results of family functioning and family satisfaction between families of hookah users and non-users, it is suggested that the use of hookah pipe is a result of poor family functioning, which in turn influences family satisfaction. However, conversely this does not suggest that poor family functioning and family satisfaction cause the use of hookah pipe.

## Limitations

Taken into consideration that this is one of the few articles which has focused on hookah use in families, there is not enough literature available to either support or counter the findings of the study. Additionally, this study did not particularly focus on the self-esteem or social skills among adolescents, which could have initiated different results in terms of their hookah use, family functioning and family satisfaction. Again, the sampling strategy used in this study is another identified limitation. This is related to two different sampling techniques used during data collection. As indicated, data collection was the result of no-one being home or individuals declining to participate in the study during the door-to-door systematic sampling process with researchers approaching every fifth house. Thus, researchers resorted to the use of convenience sampling. Using these different sampling techniques could affect the generalisability of the study. Thus, possibly findings could not be extended beyond the sample due to sampling strategies being altered during the data collection process. Moreover, the approach to sampling with the use of both convenience and systematic sampling may have introduced coverage errors and affected response rates as the sampling frame could have been restricted to only a certain portion of the population of interest. Furthermore, a contributing limitation to the study is the lack of sample size calculation before data collection to determine an adequate sample size for the study. This may have affected the study because a sufficient sample size for cross-sectional study may not have been achieved. However, a retrospective calculation was done to determine the power of the study based on the actual sample size of this study. The statistical power was deemed sufficient (>80%) to investigate family functioning and family satisfaction between hookah users and non-users.

## Recommendations

This study, together with previous research focusing on hookah, provides only a snapshot view of the problem; therefore, further research is required. As indicated in a WHO review,^9^ more research is required to understand the complexities of the reasons for using hookah. Further research should focus on the following areas: (1) Parents and their role with their children in using hookah. The research should be conducted using a developmental approach both quantitatively and qualitatively. (2) The hookah is known to be used socially and in the family, but we do not know why it is used. Perhaps, using mixed methods research, it could be possible to understand this phenomenon. Of particular concern is the inept youth with low self-esteem and social skills who may use hookah in order to be more sociable, but this area of study is unclear as there is currently no research available. Furthermore, research could also be comparatively conducted between cigarette and alcohol use. Once we are able to understand hookah and why it is used, clearer strategies could be designed.

In terms of policy, there should be much stricter regulations and implementation, as with cigarette smoking, but just focusing on the tobacco control policy alone may not be sufficient. There should also be a focus on substance abuse policy. Since hookah is used largely within the family, the application of such a policy could be challenged. Hence, the policy could specifically focus on smoke-free environments, which would include hookahs. Perhaps a review of international policies could provide insight into how to apply and adapt regulations for using hookah as well as other tobacco-free products. There should be a strong focus on reducing permissibility and exposure to children.

Prevention and intervention strategies should be designed using evidence-based approaches. Globally, this is very limited, but is increasingly required. The strategies should focus on (1) education and awareness raising using an ecological approach; (2) strengthening families, especially in terms of parenting and improving family relationships; (3) restricting or closing hookah bars, lounges or restaurants where hookahs are used; (4) providing youth with alternative means of socialising; (5) age restrictions, packaging and marketing in terms of health warnings should be as stringent as with cigarette and alcohol use; (6) considering reducing accessibility, especially for children aged less than 18 years and (7) increasing the price of all products related to hookah use.

## Conclusion

This study extends previous research regarding the role of family in hookah use. It forms part of a larger baseline study, which would ultimately design interventions and programmes to strengthen families and potentially reduce hookah use. Additionally, this study adds to our knowledge base about family’s role in hookah use which is under-researched both locally and globally. We trust that this study would provide valuable information to the City of Cape Town and could assist them in better understanding the prevalence of hookah pipe usage; the dangers associated with it in communities within the city and to inform officials when designing and implementing strategies. This would also assist in building healthier and more resilient communities. Gaps in policy in terms of regulating hookah pipe usage also call for attention.

## References

[1] WHO Study Group on Tobacco Product Regulation Advisory note: Waterpipe tobacco smoking: Health effects, research needs and recommended actions for regulators [homepage on the Internet]. 2015 [cited 2018 June 25]. Available from: http://www.who.int/tobacco/publications/prod_regulation/waterpipesecondedition/en/.

[2] Robinson JN, Wang B, Jackson KJ, Donaldson EA, Ryant CA Characteristics of hookah tobacco smoking sessions and correlates of use frequency among US adults: Findings from Wave 1 of the Population Assessment of Tobacco and Health (PATH) study. Nicotine Tob Res. 2017;20(6):731–740. 10.1093/ntr/ntx060 28340148

[3] Akl EA, Gunukula SK, Aleem S, et al The prevalence of waterpipe tobacco smoking among the general and specific populations: A systematic review. BMC Public Health. 2011;11(1):244 10.1186/1471-2458-11-244 21504559 PMC3100253

[4] Maziak W, Ward KD, Afifi Soweid RA, Eissenberg T Tobacco smoking using a waterpipe: A re-emerging strain in a global epidemic. Tob Control. 2004;13(4):327–333. 10.1136/tc.2004.008169 15564614 PMC1747964

[5] Radwan GN, Mohamed MK, El-Setouhy M, Israel E Review on water pipe smoking. J Egypt Soc Parasitol. 2003;33(3):1051–1071. 15119470

[6] Shafagoj YA, Mohammed FI Levels of maximum end-expiratory carbon monoxide and certain cardiovascular parameters following hubble-bubble smoking. Saudi Med J. 2002;23(8):953–958. 12235470

[7] Hadidi KA, Mohammed FI Nicotine content in tobacco used in hubble-bubble smoking. Saudi Med J. 2004;25(7):912–917. 15235699

[8] Roman NV, Schenck C, Jacobs L, September SJ Hookah use: Could families be a risk factor for future addiction? J Child Adolesc Subst Abuse. 2017;26(1):11–17. 10.1080/1067828X.2016.1175985

[9] Sabahy A, Divsalar K, Nakhaee N Attitude of university students towards waterpipe smoking: Study in Iran. Addict Health. 2011;3(1–2):9–14. 24494111 PMC3905517

[10] Maziak W The waterpipe: A new way of hooking youth on tobacco. Am J Addict. 2014;23(2):103–107. 10.1111/j.1521-0391.2013.12073.x 25187045 PMC4424425

[11] Baheiraei A, Sighaldeh SS, Ebadi A, Kelishadi R, Majdzadeh R The role of family on hookah smoking initiation in women: A qualitative study. Glob J Health Sci. 2015;7(5):1 10.5539/gjhs.v7n5p1 PMC4803904 26156895

[12] Daniels K, Roman NV A descriptive study of the perceptions and behaviors of waterpipe use by university students in the Western Cape, South Africa. Tob Induc Dis. 2013;11(1):4 10.1186/1617-9625-11-4 23394683 PMC3600009

[13] Senkubuge F, Ayo-Yusuf OA, Louwagie GM, Okuyemi KS Water pipe and smokeless tobacco use among medical students in South Africa. Nicotine Tob Res. 2012;14(6):755–760. 10.1093/ntr/ntr211 22039073

[14] Van der Merwe N, Banoobhai T, Gqweta A, et al Hookah pipe smoking among health sciences students. S Afri Med J. 2013;103(11):847–849. 10.7196/samj.7448 24148170

[15] Mahabee-Gittens EM, Xiao Y, Gordon JS, Khoury JC The role of family influences on adolescent smoking in different racial/ethnic groups. Nicotine Tob Res. 2011;14(3):264–273. 10.1093/ntr/ntr192 22180584 PMC3281236

[16] Biglan A, Duncan TE, Ary DV, Smolkowski K Peer and parental influences on adolescent tobacco use. J Behav Med. 1995;18(4),315–330. 7500324 10.1007/BF01857657

[17] Gutman LM, Eccles JS, Peck S, Malanchuk O The influence of family relations on trajectories of cigarette and alcohol use from early to late adolescence. J Adolesc. 2011;34(1):119–128. 10.1016/j.adolescence.2010.01.005 20129658

[18] Piko BF, Balázs MÁ Authoritative parenting style and adolescent smoking and drinking. Addict Behav. 2012;37(3):353–356. 22143001 10.1016/j.addbeh.2011.11.022

[19] Knishkowy B, Amitai Y Water-pipe (narghile) smoking: An emerging health risk behavior. Pediatrics. 2005;116(1):113–119. 10.1542/peds.2004-2173 15995011

[20] Jamil H, Janisse J, Elsouhag D, Fakhouri M, Arnetz JE, Arnetz BB Do household smoking behaviors constitute a risk factor for hookah use? Nicotine Tob Res. 2011;13(5):384–388. 21330269 10.1093/ntr/ntq249

[21] Flynn A, Tremblay PF, Rehm J, Wells S A modified random walk door-to-door recruitment strategy for collecting social and biological data relating to mental health, substance use, addiction, and violence problems in a Canadian community. Int J Alcohol Drug Res. 2013;2(2):7–16. 27279929 10.7895/ijadr.v2i2.143 PMC4894817

[22] Roman NV, Isaacs SA, Davids C, Sui XC How well are families doing? A description of family well-being in South Africa. Fam Med Community Health. 2016;4(3):9–18.

[23] Smith JR, Edland SD, Novotny TE, et al Increasing hookah use in California. Am J Public Health. 2011;101(10):1876–1879. 21852640 10.2105/AJPH.2011.300196 PMC3222344

[24] Combrink A, Irwin N, Laudin G, Naidoo K, Plagerson S, Mathee A High prevalence of hookah smoking among secondary school students in a disadvantaged community in Johannesburg. S Afr Med J. 2010;100(5):297–299. 20460022 10.7196/samj.3965

[25] Tamim H, Al-Sahab B, Akkary G, et al Cigarette and nargileh smoking practices among school students in Beirut, Lebanon. Am J Health Behav. 2007;31(1):56–63. 17181462 10.5555/ajhb.2007.31.1.56

